# Preparedness for Chagas disease spreading worldwide

**DOI:** 10.1186/s40249-020-00658-7

**Published:** 2020-04-27

**Authors:** Qin Liu, Jin Chen, Xiao-Nong Zhou

**Affiliations:** 1 National Institute of Parasitic Diseases at Chinese Center for Diseases Control and Prevention; Chinese Center for Tropical Diseases Research; WHO Collaborating Centre for Tropical Diseases, National Center for International Research on Tropical Diseases, Ministry of Science and Technology, Shanghai, 200025 People’s Republic of China; 2 School of Global Health, Chinese Center for Tropical Diseases Research, Jiatong University School of Medicine, Shanghai, 200025 People’s Republic of China

**Keywords:** Chagas disease, American trypanosomiasis, Awareness, Preparedness, Surveillance-response systems, Community-based interventions, Vector control

## Abstract

Chagas disease remains a serious problem for public health due to the high disease burden together with its global spreading patterns. However, current treatment and vector control are highly challenged by drug and insecticide resistance. Chemotherapy and vector control have been proved to be effective attempts to minimize the disease burden. Continued efforts are necessary to keep adapting the surveillance-response systems to the dynamic health systems. More attention and investments are needed to improve appropriate strategy and technology in different settings. This may be accomplished by creating effective risk early warning, addressing vulnerability and building resilience systems, implementing a vector surveillance system, as well as innovating research and technology.

## Background

Chagas disease, caused by the infection of protozoon *Trypanosoma cruzi,* is also called as American trypanosomiasis, and termed as a “silent and silenced disease”. It is one of the most common neglected tropical diseases (NTDs) but considered as the fourth most transmitted disease after malaria, tuberculosis, and schistosomiasis by the World Bank and World Health Organization (WHO) [1]. About 120 million people are at risk, and 6–8 million infected with *T. cruzi* in the Latin American and Caribbean region [2, 3]. Chagas disease once called as “the new HIV/AIDS of the Americas”, since its salient similarities existed between people living with Chagas disease and people living with HIV/AIDS [4].

**Fig. 1:**
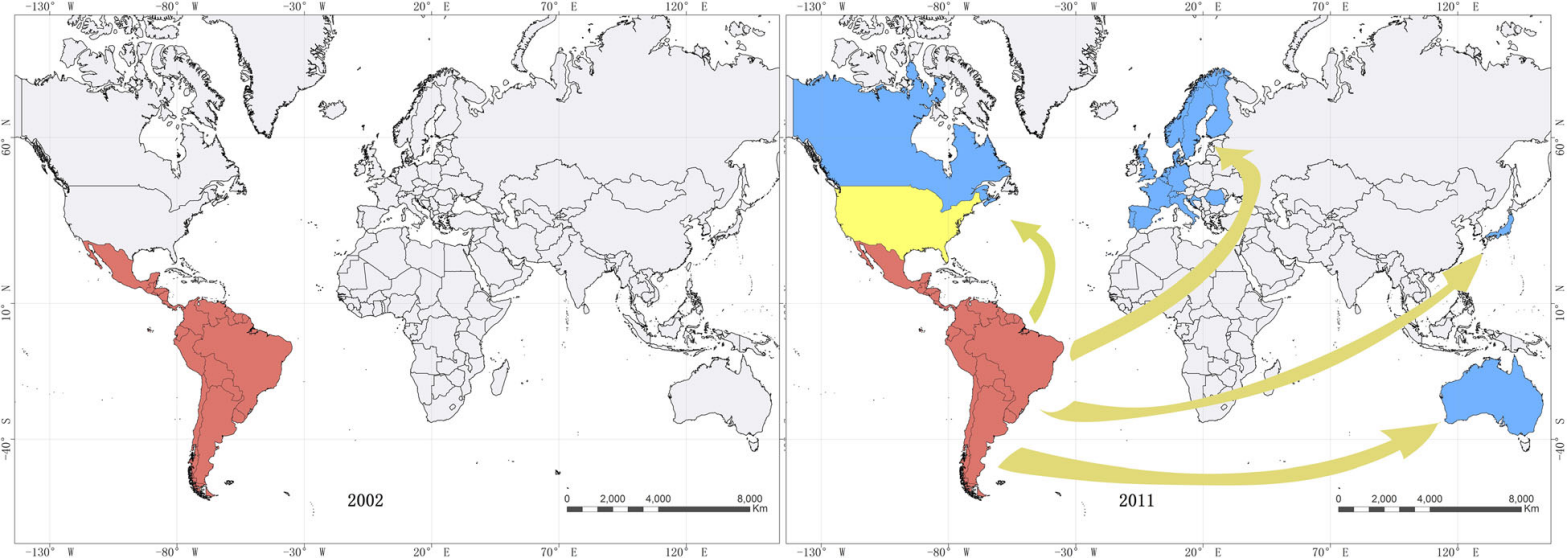
Global spreading patterns of Chagas disease in the last decade. Red: Endemic area of Chagas disease transmitted by local vectors. Yellow: Endemic area of Chagas diseases transmitted by local vector occasionally. Blue: Non-endemic areas of Chagas disease introduced by imported cases with non-vectorial transmission

With the globalization and increasing mobility during last decade, Chagas disease is spreading to non-endemic areas, including the United States of America, Canada, and many European and some Eastern Mediterranean and Western Pacific countries (Fig. 1) [1–3]. Most of cases found in non-endemic areas are infected through blood or blood product transfusion, organ transplants, mother and infant congenital transmission, consumption of contaminated food, laboratory accidents, etc. The general approach to prevent Chagas disease in non-endemic countries is performed by (i) preventing *T. cruzi* transmission by systematically screening blood used for transfusions and organs intended for transplantation; (ii) clinic diagnosis, case management, and treating patients, including infected new-borns through congenital transmission; and (iii) sharing information about Chagas disease, and training health personnel to facilitate diagnosis and medical care [1].

In order to raise public awareness of this NTD that affects mainly poor people, the 72nd World Health Assembly approved the designation of a World Chagas Disease Day in 2019. The day set on 14 April is to memorize the discovery of the disease, that the first patient, a Brazilian girl was discovered by Dr. Carlos Ribeiro Justiniano Chagas on 14 April, 1909. It is certain that setting up the World Chagas Disease Day will provide a unique opportunity to add a global voice in favour of this and other neglected diseases [5].

As an international journal addressing essential public health questions relating to infectious diseases of poverty, this journal has published several articles on epidemiology, biology, treatment and control of Chagas disease and its vectors, with its transmission as a hot topic. All results from those publications showed that transmission of Chagas disease can be effectively interrupted by controlling the main vectors and other initiatives, which have been implemented with some success. But it is still a serious problem for public health due to the threat of high disease burden in endemic regions [6], spreading to non-endemic areas [7, 8], and lack of more appropriate strategy and innovative research in resources limited settings [9]. This Editorial is to raise awareness and propose global preparedness for the world-wide spreading of Chagas disease.

**Table:** **Box 1: Transmission route of Chagas disease**

**Fig. 2:**
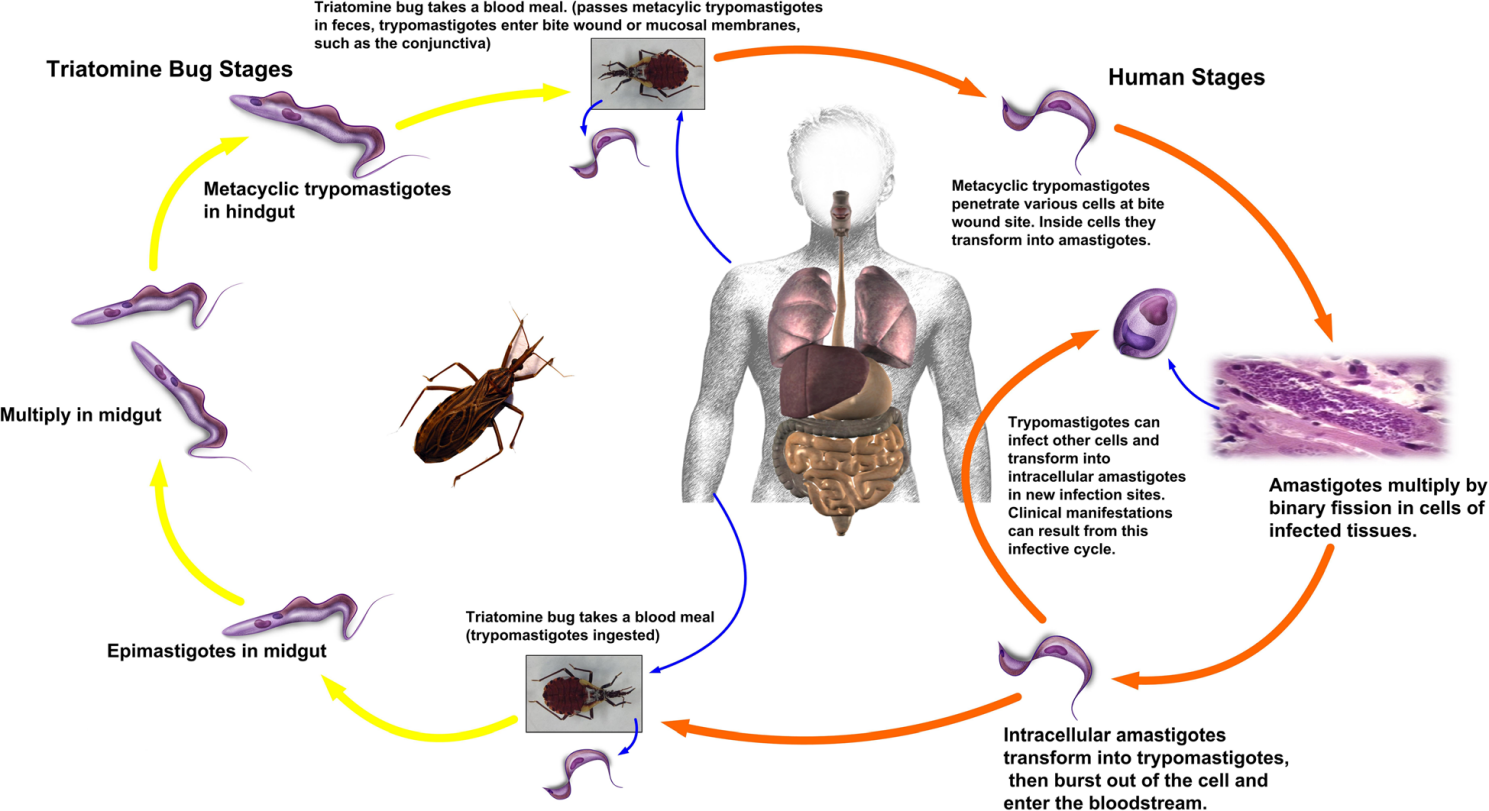
Transmission route of Chagas disease through triatomine and humans

Two phases of Chagas disease, the acute phase and the chronic phase, can be observed in the human infections. *Trypanosoma cruzi* infection is curable if treatment is initiated soon after infection. In chronic patients, antiparasitic treatment can potentially prevent or curb disease progression and prevent transmission, for instance, mother-to-child infection. The parasites, *T. cruzi*, multiply within cells in the body of the human after infection which mainly transmitted by contact with faeces/urine of infected blood-sucking triatomine bugs (Fig. 2) [1]. The bugs usually bite an exposed area of skin such as the face, hence its common name ‘kissing bug’. These bugs, especially the domestic habitated triatomine, typically live in the wall or roof cracks of homes and peridomiciliary structures, such as chicken coops, pens and warehouses, in rural or suburban areas. Vector control is the most useful method to prevent Chagas disease in Latin America.

### Community-based interventions

In addition to information dissemination, public health education and communication to raise the awareness of the disease at community level, more efforts on intervention involved by communities are also important through current health systems. In the intervention areas, chemotherapy and vector control have been proved to be effective attempts to minimize the disease burden [10]. Community-based interventions, including insecticide treated bed nets, insecticide spraying, preventive chemotherapy, and treatment, will be more effective when combining with vertical vector control programmes. This also requires high level governmental commitment along with strong partnerships among major stakeholders [11–13]. In wider context, the equitable system on social determinants of health and public health policies shall be built to ensure better health and living conditions for all individuals [6].

Community-based vector surveillance system is also workable within the health systems. Integrated with primary health intervention, an active vector surveillance system is vital to improve intervention performance and monitor the risk of imported pathogens. Continued efforts are necessary to keep adapting the surveillance system to the dynamic health systems [1, 14]. For example, application of geographical information systems and remote sensing data in the surveillance system can not only widen the knowledge of climatic, environmental, anthropogenic and biodiversity factors that influence the reduction or the re-emergence of Chagas disease, but also integrate into research and decision-making processes for mapping risks, and creating early warning systems, which will give an assistance for decision makers to better allocate limited resources [15].

## Preparedness against world-wide spreading

With the acceleration of the globalization process, the Chagas disease is also likely to occur in non-endemic countries through imported cases. It is strongly suggested that those non-endemic countries or regions with high risk of imported cases need to establish the surveillance-response systems and preparedness mechanism to monitor and control imported Chagas disease, particularly those areas where blood-sucking triatomine bugs existed, with following recommendations.

### Establishment of early warning system

The early warning system is importance to predict the potential risk areas where transmission might be emergence when imported cases occurred. The local resources to establish this kind of early warning system is needed, and allocation of these resources are based on the levels of transmission risks, so that early warning efforts are able to predominantly target to the high-risk areas. For Chagas disease, its coverage of epidemic is closely related to the transmission vector-triatomine, so the predicted risks are possible to be projected based on the survey results of vector-triatomine distribution. For instance, some researchers have extracted the climatic and environmental factors in the areas where triatomine bugs exist, created spatial and temporal stratification of the disease and population at risk [15].

### Addressing vulnerability and building resilience system

In order to respond effectively to uncertain future scenarios of vector-borne disease, more attention needs to be given to build a resilient and equitable system at present. It is recommended to adopt the community-based adaptation to foster effective organization of local people to participate in decision-making. And it can also promotes the integration of different disciplines together, and the forging of partnerships with communities, valuing local knowledge, which is an effective and integrated problem-solving approach. Under the “One Health” and “EcoHealth” concept, our Editorial highlighted the importance of building strong community-level outreach systems which can address vulnerability and build resilience of control Chagas disease and other vector-borne diseases.

### Implementing a vector surveillance-response system

The strategy to control the local transmission introduced by the imported case of Chagas disease is to interrupt vector-borne transmission, thus, it is of importance to set up a vector surveillance-response system which could minimize infection risks of Chagas disease. The monitoring and evaluation scheme for the control of native species are urgently revised internationally, so that all reports on vectors’ distribution and responses are able to be registered in a digital database worldwide. Then it can be used to describe and analyse the system performance in terms of amount of vector reports as well as rates and timeliness of responses at global level. Thus, we suggest that improvement and integration of current vector surveillance-response systems through WHO channel is an importance of creating sound opportunities to strengthen the broader and more resilient health system in resources limited settings.

## Research and development of new tools and control strategy

Current treatment methods and vector control measures are highly challenged by drug and insecticide resistance, which affects virtually all interventions currently used. More attention and investments are needed to improve appropriate strategy and technology [13]. Updated strategies in understanding epidemiological patterns and therapeutic outcome of the disease, as well as innovative research on development of more sensitive diagnostic tools and more efficient drugs, are also encouraged to sustain and scale up control successes in all endemic regions [10, 16].

## Conclusions

The improved surveillance-response systems and preparedness mechanism are of great significance to control the world-wide spread of Chagas disease, a neglected but harmful disease. The urgent needs to strengthen the health systems in both endemic and non-endemic areas are to pay more efforts on early warning of the transmission risks, addressing vulnerability and building resilience systems including a vector surveillance-response system. Innovative researches on technology development on therapeutic outcome, more sensitive diagnostic tools, and more efficient drugs, are also encouraged.

## Data Availability

Not applicable.

## Abbreviations

NTDs: Neglected tropical diseases
HIV/AIDS: human immunodeficiency virus/ Acquired immunodeficiency syndrome
WHO: World Health Organization
